# Tackling the global problem of traumatic stress in low-income countries: a pilot clinical trial comparing reconsolidation therapy to paroxetine in Nepal

**DOI:** 10.1186/s12888-021-03441-6

**Published:** 2021-09-03

**Authors:** Alain Brunet, Ram P. Sapkota, Bhushan Guragain, Jacques Tremblay, Daniel Saumier, Laurence J. Kirmayer

**Affiliations:** 1 Research Centre of the Douglas Mental Health University Institute, and Department of Psychiatry, McGill University, 6875 boulevard LaSalle, Montréal, Quebec H4H 1R3 Canada; 2 Division of Social and Transcultural Psychiatry, Global Mental Health Program, McGill University, Montréal, QC Canada; 3 Centre for Victims of Torture (CVICT), Kathmandu, Nepal; 4 Culture and Mental Health Research Unit, Lady Davis Institute, Jewish General Hospital, Montréal, QC Canada

**Keywords:** Traumatic stress, Paroxetine, Reconslidation therapy, Low– and middle–income countries, Effectiveness, Efficiency, Propranolol, Treatment, Randomized controlled trial

## Abstract

**Background:**

Traumatic stress is a global mental health problem requiring novel, easily implemented treatment solutions. We compared the effectiveness and efficiency of Reconsolidation Therapy (RT) to the well-established antidepressant paroxetine, in reducing symptoms of traumatic stress among patients from Nepal, a low-income country.

**Methods:**

Forty-six adults with posttraumatic stress disorder (PTSD) were randomized to one of two groups. The reconsolidation blocker propranolol was administered 90 min before briefly recalling a traumatic memory with a therapist, weekly for six consecutive weeks. This was compared to daily paroxetine for 26 weeks. Self-reported PTSD symptoms were assessed blindly at the 7th, 13th, and 26th weeks.

**Results:**

An intent-to-treat analysis revealed a robust pre- to post-treatment main effect (*β*_1_ = − 4.83, 95% *CI* = [− 5.66, − 4.01], *p* < .001), whereby both groups improved, with Cohen’s effect sizes of *d* = 2.34 (95% *CI* = [1.57, 3.12]) for paroxetine, and of 2.82 (95% *CI* = [1.98, 3.66]) for RT after 7 weeks, suggesting treatment effectiveness for both groups in a real-world setting. Three and six-month follow-up yielded further significant improvement in both groups, which did not differ from each other.

**Conclusion:**

RT also displayed promising efficiency, considering that it had been discontinued weeks earlier while the paroxetine treatment was continued, as recommended. RT could be taught in low-income countries as part of the local therapeutic resources to treat the core symptoms of PTSD, provided that such results are replicated on a broader scale.

**Trial registration:**

ISRCTN34308454 (11/10/2017).

## Background

Posttraumatic stress disorder (PTSD) results in great distress and impairment, and represents a major global mental health problem [1]. It remains among the most commonly reported mental disorders in conflict-affected and post-disaster settings (e.g., [2]). In Nepal, a country with a five-decade history of humanitarian interventions [3], studies find high rates of symptoms consistent with PTSD: 9–14% in the general population, 14–60% among torture survivors, 55% in former child soldiers, and 53% among internally displaced individuals [4–6]. Yet, with a few notable exceptions, evidence-based interventions for PTSD designed for individuals living in low- and middle-income countries (LMICs) are still largely lacking according to several systematic reviews (e.g., [7]). Although PTSD has been recognized as a *Grand Challenge* [8], neither the call for scale-up of evidence-based treatments for mental disorders [9] by the Movement for Global Mental Health, nor the WHO [10] flagship guidelines (the mhGAP-IG) highlight the need for the treatment of PTSD in LMICs.

Mainstream approaches to the treatment of trauma-related disorders include trauma-focused Cognitive Behavior Therapy (CBT) and Eye Movement Desensitization and Reprocessing (EMDR), as well as the use of selective serotonin reuptake inhibitors (SSRIs) like paroxetine [11]. Although efficacious [12, 13], such approaches are only moderately efficient (i.e., the capacity to reach an objective with minimum use of resources; see [14]) when duration, treatment drop-outs (up to 32% for pharmacotherapy, and 78% for psychotherapy) and relapse (up to 20% for pharmacotherapy and 50% for psychotherapy) are factored in [12, 15, 16]. Importantly, SSRIs do not offer a cure for PTSD and induce problematic side-effects. Individuals on SSRIs must remain on these medications for years in order to prevent relapse [17].

Although evidence-based psychotherapies practiced in high-income settings can be adapted to treat PTSD in LMICs (see [7]), there is a general lack of resources to implement lengthy treatments protocols that require highly qualified personnel, and intensive training and supervision (see [18]). Clearly, novel evidence-based, easily scalable treatments for PTSD are urgently needed [19, 20]. Reconsolidation Therapy is an emerging therapeutic intervention that could potentially play such a role.

### What is reconsolidation therapy?

Reconsolidation Therapy aims to weaken trauma-related emotional memories by impairing their reconsolidation. Reconsolidation theory suggests that a previously consolidated memory temporarily returns to an active state and becomes labile again when recalled under certain conditions [21, 22]. In order for the labile memory to persist in long-term memory, de novo intracellular protein synthesis is required for re-consolidation [22]. The lipophilic beta-blocker propranolol inhibits protein synthesis and weakens the memory reconsolidation process [22–24]. This pre-clinical finding was translated into a proof-of-concept study involving PTSD patients by Brunet et al. [25]. Reconsolidation blockade, or impairment, was subsequently used with success with PTSD patients in several trials (e.g., [26–28]), as well as with phobias (e.g., [29]), and addiction [28]. A meta-analysis also suggests that propranolol reduces memory reconsolidation of emotional material among healthy study participants [30].

Reconsolidation Therapy (RT) is a promising, simple, and easy to learn protocolized treatment for PTSD [30] embodying the principles of reconsolidation theory. However, its effectiveness and efficiency, i.e., the capacity to achieve a therapeutic goal with a lesser dose of treatment or while consuming less (human or material) resources, remains poorly documented in non-Western, low-income settings. This is of importance considering that most trauma due to humanitarian emergencies occur in non-Western countries [31, 32] where formal mental health resources are scarce.

### Main hypothesis

This pilot randomized controlled trial investigated the (real-world) effectiveness and efficiency of Reconsolidation Therapy compared to paroxetine in reducing symptoms of PTSD. In light of previous literature [26, 30, 33, 34], we hypothesized that: (i) effectiveness would be observed for both interventions in reducing PTSD symptoms after 7 weeks of treatment; and that (ii) RT would be more efficient than paroxetine, i.e., achieve a similar or greater treatment effect but with a smaller dose of treatment [14], over the course of this 26-weeks study.

## Methods

### Participants and setting

Participants were recruited in 01/04/2013 and 01/02/2014 through a government-run health post in the village of Manpur (Dang district) in rural Nepal. The estimated population of the Dang district was 552,583, constituting several linguistic and ethnic groups. This district was severely affected during the Maoist armed conflict in Nepal. Civilians sustained acts of extreme violence (e.g., abduction, killing, torture, sexual abuse) from warring parties (see [4, 35]). An epidemiological survey found high rates of anxiety, depression, PTSD and psychosocial problems in Dang [4]. See Table 1 for the participants’ sociodemographic characteristics.

**Table 1:** Participant characteristics at pre-treatment per group

Characteristics at baseline	Paroxetine			Reconsolidation		
	*n*	*M*	*SD*	*n*	*M*	*SD*
Age	23	40.30	11.85	23	39.78	10.95
PTSD symptoms (PCL)	23	65.56	7.83	23	64.73	7.76
Anxio-depressive symptoms (HSCL-25)	23	3.21	0.40	23	3.18	0.41
	*n*	%		*n*	%	
**Gender**^**1**^						
Male	4	17.39		11	47.83	
Female	19	82.61		12	52.17	
**Education**						
Illiterate	14	60.87		8	34.78	
Primary	6	26.09		10	43.48	
Secondary (8–10 years) or more	3	13.04		5	21.74	
**Profession**						
Service	1	4.35		2	8.70	
Homemaker	7	30.43		4	17.39	
Agriculture	13	56.52		15	65.21	
Other	2	8.70		2	8.70	
**Self-reported socio-economic status**						
Lower-class	14	60.87		14	60.87	
Middle-class	9	39.13		9	39.13	
**Ethnicity**						
Brahman	2	8.70		0	0	
Chhetri	8	34.78		11	47.83	
Janajati	8	34.78		7	30.43	
Dalit	5	21.74		5	21.74	
**Marital status**						
Never married	2	8.70		3	13.04	
Married	20	86.95		19	82.61	
Single (divorced or widowed)	1	4.35		1	4.35	

^1^Gender is the only significant between-groups difference, *X*^2^ = (1, *N* = 46) = 4.8, *p* < .05

### Study design

This retrospectively registered (ISRCTN34308454) randomized controlled trial used a 1:1 two-arms block design, and a block size of four [36]. Participants were assessed on the first treatment day (baseline) immediately before randomization, and again blindly 7, 13 and 26 weeks later at the health post by a trained health assistant. All procedures contributing to this work comply with the ethical standards of the relevant national and institutional committees on human experimentation and with the Helsinki Declaration of 1975, as revised in 2008. This trial was approved by Health Canada, by the Ethics Review Board of the Douglas Institute (ref: 12/4), and by an ad-hoc ethics committee from Tribhuvan University Teaching Hospital in Kathmandu, Nepal. To circumvent literacy issues, participants were free to choose between oral (video-recorded) or written informed consent. Data compilation and analysis was slowed due to the 2015 earthquakes which affected the offices of our partner institution, the Centre for Victims of Torture (CVICT) in Nepal.

The inclusion criteria comprised the following: consented treatment-seeker aged 25–65; experienced a traumatic event, that is, the person experienced or witnessed an event that involved actual or threatened death or serious injury, or a threat to the physical integrity of self or others > 6 months ago [37]; met the DSM-IV-TR diagnostic criteria for PTSD. The exclusion criteria were: having a basal heart rate < 60 bpm; a resting systolic blood pressure < 100 mmHg; a self-reported PTSD symptom score < 50 (see instruments section below); active suicidal ideation; a history of congestive heart failure, diabetes, chronic bronchitis, emphysema, or asthma; a medical morbidity requiring immediate treatment; a previous adverse reaction to a β-blocker or SSRI; a woman of child-bearing potential not using an acceptable contraceptive method, or pregnant or breast-feeding; a lifetime diagnosis of psychotic or bipolar disorder, cognitive problems, alcohol/substance dependence; using a medication that may interact adversely with propranolol or paroxetine.

### Study medications

Participants from the RT group received 1 mg/kg of propranolol once a week, for 6 consecutive weeks, administered with a snack in order to increase absorption [38]. Propranolol is among the World Health Organization (WHO) list of essential and safe medications that are used worldwide [39].

Paroxetine, a widely prescribed lipophilic amine and a high affinity SSRI almost completely absorbed by the gastrointestinal tract after oral administration, is the recommended pharmacological treatment for adults with PTSD in the US [11]. We used the product monograph’s recommended therapeutic dose of 20 mg/day once daily for 13 weeks, and then 10 mg daily for 13 more weeks. The paroxetine capsules were dispensed at weekly divests to the health post and self-administered at home every day. To ensure treatment adherence, transportation costs from and to the health post were reimbursed. All medications were packaged by the Douglas Institute pharmacy according to Canadian regulations.

### Outcome measures

Symptom severity was assessed with the self-report PTSD Checklist (PCL [6];). Psychiatric comorbidity (i.e., anxiety and depression) was assessed with the self-report Hopkins Symptoms Checklist (HSCL-25 [6];). A DSM-IV-TR PTSD diagnosis was determined using the Mini International Neuropsychiatric Interview (MINI 5.0.0 [40];). The MINI was translated in Nepali and culturally adapted following the guidelines of Van Ommeren et al. [41]. Briefly, the translated measure was presented to the clinical staff of CVICT who provided feedback. The revised version was back translated into English and compared to the original version by a native English speaker. The Nepali translation was field tested among our case finders.

### Treatment and procedure

**Fig. 1:**
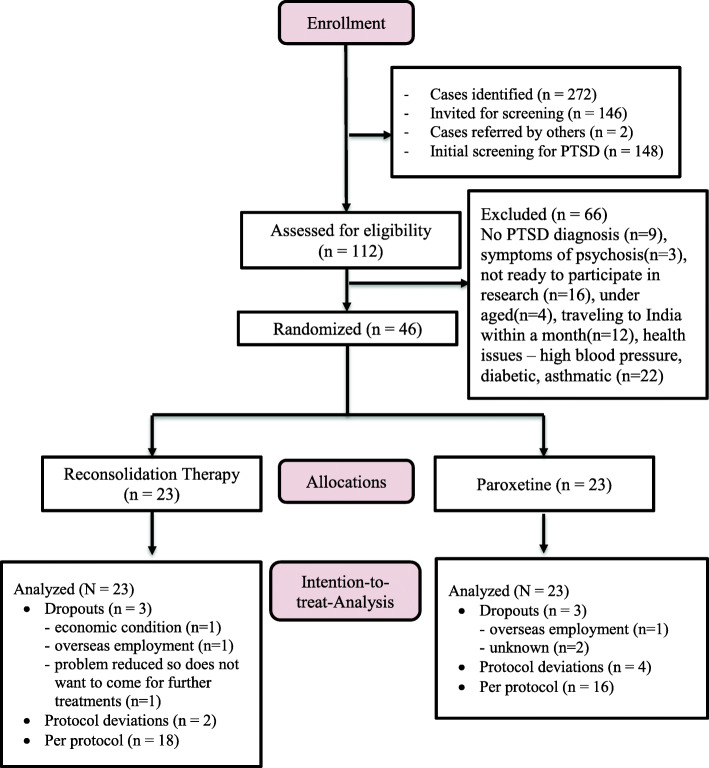
Participants’ flow diagram

Four experienced psychosocial counselors, working for CVICT in Dang district, were trained as case finders. Candidate participants were identified by word of mouth, visited, and screened at home by the case finders. Seemingly eligible candidate participants were invited to the health-post for a complete assessment by a trained medical doctor (B.G.). Qualifying participants were immediately randomized to one of two treatment arms using a 1:1 ratio and a block size of four [36]. The randomization list was stored in a password-protected computer at CVICT’s office in Kathmandu. An assistant at CVICT communicated group assignment blindly over the phone to the medical doctor located in Dang. See Fig. 1 for the participants’ inclusion flow chart.

Two female nurses were formally trained by A.B. to use Reconsolidation Therapy™. The intervention consisted in the patient writing/reading, or orally narrating the index traumatic event in the first person, present tense for 10–15 min to the nurse. R.P.S. acted as interpreter and cultural advisor. The nurses practiced the treatment for 6 weeks with six pilot participants (not included in the study trial). A.B. and R.P.S. provided corrective feedback 6 weeks after the initial training was completed. The treatment sessions were videotaped and 50% of sessions were rated by a panel (A.B. and R.P.S.) using an adherence checklist devised for this study (available from the first author). Adherence was deemed good to excellent, with some minor departures (e.g., narratives verbalized in the past instead of the present tense).

Trauma reactivation was conducted as described by Brunet et al. [25, 26], at the health post once a week for six consecutive weeks. Briefly, a nurse administered the propranolol under the supervision of a medical doctor. Vital signs were monitored every 30 min for 90 min, after which the videotaped narration began. The blind assessments took place at the health post at 7th, 13th, and 26th weeks.

### Statistical analyses

Analyses were conducted with SPSS 22.0 for Windows (IBM, Armonk, NY). The treatment groups were compared twice, using two-sided tests with alpha set at .05: 1) using an intention-to-treat (ITT) sample, for which data were imputed using the Expectation-Maximization (EM) method [42] such that all randomized participants were included, and 2) using a per-protocol (PP) sample, for which unimputed data were used and the sample was restricted to treatment completers. To determine if pre- vs. post-treatment scores differed across groups, mixed models were computed with all data available across the seven (weekly) assessment points. Random effects for the intercept and time variables were tested and included in the model to take into account the correlated nature of the data. The fixed-effect model included group, time and their interaction, and estimates of fixed effects were computed. PCL data were missing for 3 participants in each group for five treatment sessions and post-treatment assessment. Since mixed-model analysis can handle missing data [43], imputation prior to analysis was not necessary for the PP sample.

At the week 7 and week 26 follow-up, there were 6 (3 in each group) and 12 (7 in the paroxetine group and 5 in the RT group) participants with PCL data missing, respectively, because of participant dropout. There were no significant sociodemographic differences between the dropouts and treatment completers. Since gender distribution differed across groups, to determine if adjusting the model for gender changed the results, the mixed-model analyses were recomputed including gender as a covariate. Effect sizes (Cohen’s d [44];) for the difference between pre- and post-treatment measures, as well as week 7 and week 13, were calculated for both samples.

## Results

A total of 46 individuals suffering from chronic PTSD were randomized to paroxetine (*n* = 23) and reconsolidation therapy (*n* = 23) groups (see Fig. 1). Of the 46 randomized, 40 (87.0%) completed the 6-week-long treatment with some protocol deviation; 34 (73.9%) participants (16 in paroxetine and 18 in reconsolidation therapy group) completed the treatment per-protocol; 6 participants (13.0%; 3 in each treatment arm) dropout from the study because of economic reasons (not mentioned), found employment overseas, or because their problems/symptoms diminished, and they did not want to come for further treatment.

Table 1 shows the demographic and clinical characteristics of the participants at baseline. There were no significant between-group sociodemographic differences at baseline, except for gender, *X*^*2*^ = (1, *N* = 46) = 4.8, *p* < .05. There were 15 males and 31 females included in the study (see Table 1).

The patient’s index event occurred mostly (87%) during the armed conflict in Nepal (1996–2006) and involved torture, having a family member killed or kidnapped, being involved in armed conflict, having to flee/hide so as not to be killed, being attacked physically or with a weapon, and being ill-treated in prison.

**Fig. 2:**
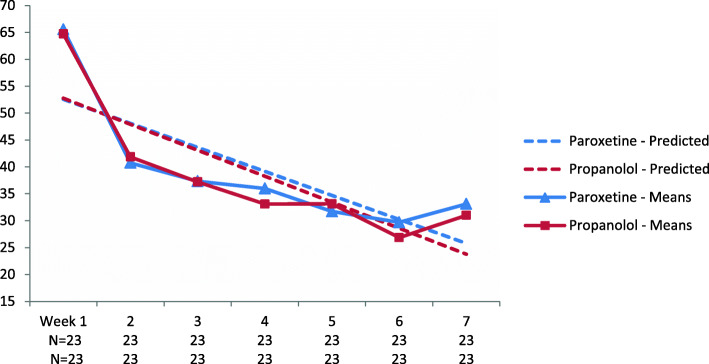
Reduction in self-reported PTSD symptoms across the first seven weeks

As hypothesized, a mixed model comparing the effects of Reconsolidation Therapy to paroxetine in the ITT group yielded a non-significant interaction effect (*β*_1_ = 0.34, 95% *CI* = [− 0.80, 1.53], *p* = .537), suggesting that the change in self-reported PTSD symptoms over 7 weeks was not different across groups (see Fig. 2). However, a very large reduction in PTSD symptoms was observed (*β*_1_ = − 4.83, 95% *CI* = [− 5.66, − 4.01], *p* < .001) in both groups, as illustrated in Fig. 2. Similar results were obtained in the PP analysis, yielding a non-significant group by time interaction (*F* [1, 202] = 0.051, *p* = .821), and a main effect for time (*F* [1, 202] = 273.236, *p* < .001).

Only five individuals in the paroxetine group and two in the reconsolidation therapy group scored above the PCL cut-off score of 50 at the end of treatment (week 7). The pre- vs. post-treatment effect sizes were *d* = 2.34 (95% *CI* = [1.57, 3.12]) and 2.82 (95% *CI* = [1.98, 3.66]) for paroxetine and reconsolidation therapy, respectively. Adjusting for gender effects did not change the results (not shown).

### Long-term treatment effect on PTSD symptoms

Paired-sample *t*-tests showed large significant decreases from week 7 to 13 in both the paroxetine (*d* = 0.44, *p* = .011) and reconsolidation therapy (*d* = 0.38, *p* = 0.012) groups, as well as from week 7 to week 26 (*d* = 0.81, *p* < .001; *d* = 0.81, *p* < .001, respectively). Further, none of the participants scored above the cut-off score on the PCL.

### Adverse effects

The trauma reactivation procedure was well tolerated, as further suggested by the low drop-out rate (13%) similar in both groups. In the paroxetine group, six individuals reported transient adverse physical effects: headaches and dizziness (*n* = 2), pain in epigastric region, physical weakness, an itchy throat, and nausea. One individual in the reconsolidation therapy complained of a mild pain around the heart during session two. For all cases, a medical examination did not reveal any problem. Symptoms were judged as non-serious, and treatment was pursued.

## Discussion

Six brief once-a-week sessions of RT performed by locally trained nurses were found to be non-inferior to 26 weeks of paroxetine treatment among a sample of rural Nepali men and women. The intervention yielded very large positive treatment effect in both groups. These results were maintained at both follow-ups. These results are consistent with a recently published placebo-controlled randomized trial by Brunet and colleagues [27] which showed a very large effect sizes of *d* = 2.74 for RT in a North American sample. The treatment effects for paroxetine were also comparable to those found in other studies, with *d* = 1.5–2.2 [33, 34].

Although the effect sizes were substantial in both groups, it is important to note that in the paroxetine group, this was not a sustained post-treatment effect but actually an active treatment effect, as participants continued to use the medication throughout their enrolment in the study. This points toward the possibility that the time-limited RT may have greater efficiency than paroxetine treatment. Although RT yielded treatment effects comparable to those produced by paroxetine, these were obtained using only six propranolol pills taken 90 min before a brief trauma reactivation session, with very few side effects. Further, the RT treatment effects persisted for at least 6 months after the treatment was terminated. RT was well-accepted by all the study participants. Treatment guidelines do not recommend stopping an SSRI after 6 weeks. In fact, treatment guidelines suggest taking an SSRI for at least 12 months, and then once improvement is achieved to prolong treatment for another 12 months so as to prevent the risk of relapse [45]. Of note, no relapse was observed at the 6-month follow-up in the group receiving RT.

Recall of trauma memories is at the foundation of RT. While previous studies conducted in Nepal suggested that experience and expression of trauma is stigmatized in Nepali culture (e.g., [46]), we encountered no such issues in trauma reactivation, even among some female participants who presented issues of sexual abuse and marital rape as an index traumatic experience. RT was well accepted as a treatment by all the study participants.

For obvious logistical reasons, this study could not be conducted in a double-blind manner, but this is the case for most psychotherapy research. Further, we cannot determine the proportion of improvement in both groups attributable to nonspecific effects of enrolment in a trial or clinical attention because we did not use a placebo (or sham) control group. We opted to offer two active forms of treatment to enhance feasibility and because not all participants in this rural part of Nepal were familiar with placebo trials. However, both RT and the SSRIs have reliably outperformed the placebo condition in other publications (e.g., [27]). There was a statistical trend whereby more female participants ended up in the paroxetine group than in the RT group. This trend may have increased the effect size of paroxetine, as women are more responsive to SSRIs than men [47].

## Conclusion

This study suggests that evidence-based RT can be successfully taught in culturally diverse low-resource settings and that individuals with chronic symptoms of PTSD are amenable to undergo RT and derive substantial benefits in just a few weeks. To generalize the findings of this study to other low-resource settings, the study needs need to be replicated in larger samples across diverse cultural contexts. If replicated, the reconsolidation therapy could offer a cost-effective solution for practitioners in LMICs searching for an easy to learn, brief evidence-based translational treatment for PTSD and other event-related anxiety disorders.

## Data Availability

The datasets used and/or analysed during the current study are available from the corresponding author on reasonable request.

## Abbreviations

CBT: Cognitive Behaviour Therapy
CVICT: Centre for Victims of Torture (Nepal)
DSM-IV-TR: Diagnostic and Statistical Manual of Mental Disorders, Fourth Edition, Text Revision
EM: Expectation Maximization
EMDR: Eye Movement Desensitization and Reprocessing
HSCL-25: Hopkins Symptom Checklist-25
ITT: Intention-To-Treat
LMICs: Lower- and Middle-Income Countries
MINI 5.0.0: Mini-International Neuropsychiatric Interview, Version 5.0.0
mhGAP-IG: Mental Health Gap Action Programme Intervention Guide
PCL: PTSD CheckList
PP: Per-Protocol
PTSD: Posttraumatic Stress Disorder
RT: Reconsolidation Therapy
SPSS: Statistical Package for the Social Sciences
SSRIs: Selective Serotonin Reuptake Inhibitors
WHO: World Health Organization

## References

[1] Kessler RC, Aguilar-Gaxiola S, Alonso J, Benjet C, Bromet EJ, Cardoso G (2017). Trauma and PTSD in the WHO world mental health surveys. Eur J Psychotraumatol.

[2] Steel Z, Chey T, Silove D, Marnane C, Bryant RA, Van Ommeren M (2009). Association of torture and other potentially traumatic events with mental health outcomes among populations exposed to mass conflict and displacement: a systematic review and meta-analysis. JAMA.

[3] Chase LE, Sapkota RP, Crafa D, Kirmayer LJ (2018). Culture and mental health in Nepal: an interdisciplinary scoping review. Glob Ment Health (Camb).

[4] Luitel NP, Jordans MJ, Sapkota RP, Tol WA, Kohrt BA, Thapa SB (2013). Conflict and mental health: a cross-sectional epidemiological study in Nepal. Soc Psychiatry Psychiatr Epidemiol.

[5] Tol WA, Kohrt BA, Jordans MJ, Thapa SB, Pettigrew J, Upadhaya N (2010). Political violence and mental health: a multi-disciplinary review of the literature on Nepal. Soc Sci Med.

[6] Thapa SB, Hauff E (2005). Psychological distress among displaced persons during an armed conflict in Nepal. Soc Psychiatry Psychiatr Epidemiol.

[7] Morina N, Malek M, Nickerson A, Bryant RA (2017). Psychological interventions for post-traumatic stress disorder and depression in young survivors of mass violence in low- and middle-income countries: meta-analysis. Br J Psychiatry.

[8] Collins PY, Patel V, Joestl SS, March D, Insel TR, Daar AS, Anderson W, Dhansay MA, Phillips A, Shurin S, Walport M, Ewart W, Savill SJ, Bordin IA, Costello EJ, Durkin M, Fairburn C, Glass RI, Hall W, Huang Y, Hyman SE, Jamison K, Kaaya S, Kapur S, Kleinman A, Ogunniyi A, Otero-Ojeda A, Poo MM, Ravindranath V, Sahakian BJ, Saxena S, Singer PA, Stein DJ, Scientific Advisory Board and the Executive Committee of the Grand Challenges on Global Mental Health (2011). Grand challenges in global mental health. Nature.

[9] Chisholm D, Flisher AJ, Lund C, Patel V, Saxena S, Thornicroft G (2007). Scale up services for mental disorders: a call for action. Lancet.

[10] mhGAP intervention Guide – Version 2.0 for mental, neurological and substance user disorders in non-specialized health settings . https://www.who.int/mental_health/mhgap/mhGAP_intervention_guide_02/en/. 2016 Cited March 23, 2019. 27786430

[11] Clinical Practice Guideline for the Treatment of Posttraumatic Stress Disorder (PTSD) [Internet]. https://www.apa.org/ptsd-guideline/ptsd.pdf. 2017 Cited March 23, 2019.

[12] Bradley R, Greene J, Russ E, Dutra L, Westen D (2005). A multidimensional meta-analysis of psychotherapy for PTSD. Am J Psychiatry.

[13] Hoskins M, Pearce J, Bethell A, Dankova L, Barbui C, Tol WA, van Ommeren M, de Jong J, Seedat S, Chen H, Bisson JI (2015). Pharmacotherapy for post-traumatic stress disorder: systematic review and meta-analysis. Br J Psychiatry.

[14] Andrews G (1999). Efficacy, effectiveness and efficiency in mental health service delivery. Aust N Z J Psychiatry.

[15] Batelaan NM, Bosman RC, Muntingh A, Scholten WD, Huijbregts KM, van Balkom A (2017). Risk of relapse after antidepressant discontinuation in anxiety disorders, obsessive-compulsive disorder, and post-traumatic stress disorder: systematic review and meta-analysis of relapse prevention trials. BMJ.

[16] Goetter EM, Bui E, Ojserkis RA, Zakarian RJ, Brendel RW, Simon NM (2015). A systematic review of dropout from psychotherapy for posttraumatic stress disorder among Iraq and Afghanistan combat veterans. J Trauma Stress.

[17] Katzman MA, Bleau P, Blier P, Chokka P, Kjernisted K, Van Ameringen M (2014). Canadian clinical practice guidelines for the management of anxiety, posttraumatic stress and obsessive-compulsive disorders. BMC Psychiatry.

[18] Van Ginneken N, Tharyan P, Lewin S, Rao GN, Meera S, Pian J (2013). Non-specialist health worker interventions for the care of mental, neurological and substance-abuse disorders in low-and middle-income countries. Cochrane Database Syst Rev.

[19] Koirala R, Soegaard EGI, Thapa SB (2017). Updates on pharmacological treatment of post-traumatic stress disorder. J Nepal Med Assoc.

[20] Purgato M, Abdulmalik JO, Prina E, Ceccarelli C, Tol WA, Ginneken N (2021). Primary-level and community worker interventions for the prevention of mental disorders and the promotion of well-being in low-and middle-income countries. Cochrane Database Syst Rev.

[21] Przybyslawski J, Sara SJ (1997). Reconsolidation of memory after its reactivation. Behav Brain Res.

[22] Nader K, Schafe GE, Le Doux JE (2000). Fear memories require protein synthesis in the amygdala for reconsolidation after retrieval. Nature..

[23] Cahill L, Pham CA, Setlow B (2000). Impaired memory consolidation in rats produced with beta-adrenergic blockade. Neurobiol Learn Mem.

[24] Sara SJ (2000). Retrieval and reconsolidation: toward a neurobiology of remembering. Learn Mem.

[25] Brunet A, Orr SP, Tremblay J, Robertson K, Nader K, Pitman RK (2008). Effect of post-retrieval propranolol on psychophysiologic responding during subsequent script-driven traumatic imagery in post-traumatic stress disorder. J Psychiatr Res.

[26] Brunet A, Poundja J, Tremblay J, Bui E, Thomas E, Orr SP, Azzoug A, Birmes P, Pitman RK (2011). Trauma reactivation under the influence of propranolol decreases posttraumatic stress symptoms and disorder: 3 open-label trials. J Clin Psychopharmacol.

[27] Brunet A, Saumier D, Liu A, Streiner DL, Tremblay J, Pitman RK (2018). Reduction of PTSD symptoms with pre-reactivation propranolol therapy: a randomized controlled trial. Am J Psychiatry.

[28] Lonergan M, Saumier D, Tremblay J, Kieffer B, Brown TG, Brunet A (2016). Reactivating addiction-related memories under propranolol to reduce craving: a pilot randomized controlled trial. J Behav Ther Exp Psychiatry.

[29] Sevenster D, Beckers T, Kindt M (2013). Prediction error governs pharmacologically induced amnesia for learned fear. Science.

[30] Lonergan M, Olivera-Figueroa LA, Pitman RK, Brunet A (2013). Propranolol's effects on the consolidation and reconsolidation of long-term emotional memory in healthy participants: a meta-analysis. J Psychiatry Neurosci.

[31] UNOCHA. Global humanitarian overview 2020. Geneva; 2020. https://www.unocha.org/global-humanitarian-overview-2021.

[32] Papola D, Purgato M, Gastaldon C, Bovo C, Ommeren M, Barbui C (2020). Psychological and social interventions for the prevention of mental disorders in people living in low-and middle-income countries affected by humanitarian crises. Cochrane Database Syst Rev.

[33] Kim Y, Asukai N, Konishi T, Kato H, Hirotsune H, Maeda M, Inoue H, Narita H, Iwasaki M (2008). Clinical evaluation of paroxetine in post-traumatic stress disorder (PTSD): 52-week, non-comparative open-label study for clinical use experience. Psychiatry Clin Neurosci.

[34] Lee P, Shu L, Xu X, Wang CY, Lee MS, Liu CY (2007). Once-daily duloxetine 60 mg in the treatment of major depressive disorder: multicenter, double-blind, randomized, paroxetine-controlled, non-inferiority trial in China, Korea, Taiwan and Brazil. Psychiatry Clin Neurosci.

[35] Kienzler H, Sapkota RP (2020). The long-term mental health consequences of torture, loss, and insecurity: a qualitative study among survivors of armed conflict in the Dang District of Nepal. Front Psychiatry..

[36] Fleiss JL. The design and analysis of clinical experiments: John Wiley & Sons, Inc.; 1986.

[37] American Psychiatric Association. Diagnostic and statistical manual of mental disorders, text revision (DSM-IV-TR): American Psychiatric Association; 2000.

[38] Melander A (1978). Influence of food on the bioavailability of drugs. Clin Pharmacokinet.

[39] World Health Organization (2019). World Health Organization model list of essential medicines: 21st list 2019.

[40] Sheehan DV, Lecrubier Y, Sheehan KH, Amorim P, Janavs J, Weiller E (1998). The Mini-International Neuropsychiatric Interview (M.I.N.I.): the development and validation of a structured diagnostic psychiatric interview for DSM-IV and ICD-10. J Clin Psychiatry.

[41] Van Ommeren M, Sharma B, Thapa S (1999). Ramesh Makaju, Prasain D, Bhattarai R, et al. preparing instruments for transcultural research: use of the translation monitoring form with Nepali-speaking Bhutanese refugees. Transcult Psychiatry.

[42] Little RJ, Rubin DB (2002). Statistical analysis with missing data.

[43] Twisk J, de Boer M, de Vente W, Heymans M (2013). Multiple imputation of missing values was not necessary before performing a longitudinal mixed-model analysis. J Clin Epidemiol.

[44] Cohen J (1988). Statistical power analysis for the behavioral sciences.

[45] Dua T, Barbui C, Clark N, Fleischmann A, Poznyak V, van Ommeren M, Yasamy MT, Ayuso-Mateos JL, Birbeck GL, Drummond C, Freeman M, Giannakopoulos P, Levav I, Obot IS, Omigbodun O, Patel V, Phillips M, Prince M, Rahimi-Movaghar A, Rahman A, Sander JW, Saunders JB, Servili C, Rangaswamy T, Unützer J, Ventevogel P, Vijayakumar L, Thornicroft G, Saxena S (2011). Evidence-based guidelines for mental, neurological, and substance use disorders in low-and middle-income countries: summary of WHO recommendations. PLoS Med.

[46] Kohrt BA, Hruschka DJ (2010). Nepali concepts of psychological trauma: the role of idioms of distress, ethnopsychology and ethnophysiology in alleviating suffering and preventing stigma. Cult Med Psychiatry.

[47] LeGates TA, Kvarta MD, Thompson SM (2019). Sex differences in antidepressant efficacy. Neuropsychopharmacology.

